# Activin-A Induces Early Differential Gene Expression Exclusively in Periodontal Ligament Fibroblasts from *Fibrodysplasia Ossificans Progressiva* Patients

**DOI:** 10.3390/biomedicines9060629

**Published:** 2021-06-01

**Authors:** Ton Schoenmaker, Michal Mokry, Dimitra Micha, Coen Netelenbos, Nathalie Bravenboer, Marjolijn Gilijamse, E. Marelise W. Eekhoff, Teun J. de Vries

**Affiliations:** 1 Department of Periodontology, Academic Centre for Dentistry Amsterdam (ACTA), University of Amsterdam and Vrije Universiteit, 1012 Amsterdam, The Netherlands; teun.devries@acta.nl; 2 Division Laboratory, Pharmacy and Biomedical Genetics, UMC Utrecht, 3508 Utrecht, The Netherlands; m.mokry@umcutrecht.nl; 3 Department of Clinical Genetics, Amsterdam Movement Sciences, Amsterdam UMC, Vrije Universiteit, 1012 Amsterdam, The Netherlands; d.micha@amsterdamumc.nl; 4 Department of Internal Medicine Section Endocrinology, Amsterdam Movement Sciences, Amsterdam UMC, Vrije Universiteit Amsterdam, 1012 Amsterdam, The Netherlands; c.netelen@amsterdamumc.nl (C.N.); emw.eekhoff@amsterdamumc.nl (E.M.W.E.); 5 Department of Clinical Chemistry, Amsterdam Movement Sciences, Vrije Universiteit Amsterdam, 1012 Amsterdam, The Netherlands; n.bravenboer@amsterdamumc.nl; 6 Department of Oral and Maxillofacial Surgery and Oral Pathology, Amsterdam UMC, Vrije Universiteit, 1012 Amsterdam, The Netherlands; m.gilijamse@amsterdamumc.nl

**Keywords:** *fibrodysplasia ossificans progressiva*, Activin-A, periodontal ligament fibroblasts, RNA sequencing, heterotopic ossification

## Abstract

*Fibrodysplasia Ossificans Progressiva* (FOP) is a rare genetic disease characterized by heterotopic ossification (HO). It is caused by mutations in the Activin receptor type 1 (*ACVR1*) gene, resulting in enhanced responsiveness to ligands, specifically to Activin-A. Though it has been shown that capturing Activin-A protects against heterotopic ossification in animal models, the exact underlying mechanisms at the gene expression level causing ACVR1 R206H-mediated ossifications and progression are thus far unknown. We investigated the early transcriptomic changes induced by Activin-A of healthy control and patient-derived periodontal ligament fibroblasts (PLF) isolated from extracted teeth by RNA sequencing analysis. To study early differences in response to Activin-A, periodontal ligament fibroblasts from six control teeth and from six FOP patient teeth were cultured for 24 h without and with 50 ng/mL Activin-A and analyzed with RNA sequencing. Pathway analysis on genes upregulated by Activin-A in FOP cells showed an association with pathways involved in, among others, Activin, TGFβ, and BMP signaling. Differential gene expression induced by Activin-A was exclusively seen in the FOP cells. Median centered supervised gene expression analysis showed distinct clusters of up- and downregulated genes in the FOP cultures after stimulation with Activin-A. The upregulated genes with high fold changes like *SHOC2*, *TTC1*, *PAPSS2*, *DOCK7*, and *LOX* are all associated with bone metabolism. Our open-ended approach to investigating the early effect of Activin-A on gene expression in control and FOP PLF shows that the molecule exclusively induces differential gene expression in FOP cells and not in control cells.

## 1. Introduction

*Fibrodysplasia ossificans progressiva* (FOP) is a rare, one-in-two-million-occurring, autosomal dominant genetic disease characterized by progressive heterotopic bone formation (HO) where especially muscles, tendons, and ligaments are converted into bone [1,2,3]. The clinical manifestation of the heterotopic ossification is rather diverse. HO can occur after a flare up, during inflammation, after injury, or even spontaneously. In some patients, HO is more progressive than in others, and episodes exist with complete absence of HO formation [4]. Because of these differences in clinical manifestations, cell biological approaches that can shed light on the biochemical events that precede heterotopic ossification are of great importance as a prelude to therapy. Over the past decade, our understanding of the pathogenesis of the disease has improved considerably. The causative mutation in the gene encoding the Activin Receptor Type I receptor (ACVR1) was identified by the group of Shore et al. [5]. The most frequent R206H mutation changes an arginine to a histidine in the glycine–serine rich cytoplasmic domain of this bone morphogenetic protein (BMP) type I receptor [2,5]. This amino acid change makes the receptor more sensitive to BMP signaling and simultaneously results in decreased binding of the receptor’s natural inhibitor, FKBP12, likely resulting in leaky signaling of the receptor through SMADs, the main signal transducers of the transforming growth factor-beta (TGF-β) family [6,7,8,9,10]. More recently, the discovery by two independent groups that Activin-A can also function as an activator of the mutated ACVR1 [11,12] has given great impulse to the FOP research community, since now it is conceivable to specifically prevent heterotopic bone formation by inhibiting Activin-A. Under normal circumstances, Activin-A signals mainly via ACVR1B receptor complexes through SMAD 2/3 phosphorylation [13,14,15]. Upon binding to ACVR1, it normally inhibits SMAD 1/5/9 signaling and subsequent osteogenic differentiation [16], but, as mentioned before, when Activin-A binds to the mutated ACVR1, it activates SMAD1/5/9 phosphorylation and subsequent osteogenic differentiation [11,12]. This was demonstrated by Hatsell et al. in their FOP mouse model, but also in the currently conducted LUMINA-I trial investigating the Activin-A blocking antibody Garetosmab, which has revealed promising results in the prevention of newly formed HO [17,18]. Even though it has by now been clearly shown that capturing Activin-A protects against heterotopic ossification, this knowledge is mainly limited to mouse models for FOP. Moreover, the exact underlying mechanism at the gene expression level causing ACVR1 R206H-mediated ossification and progression of the disease is unknown. Thus, knowledge of the downstream transcriptomic changes after Activin-A-ACVR1 R206H binding using open-ended approaches such as RNA sequencing is warranted.

One of the challenges that FOP research faces is the lack of primary cells from patients, since surgical removal of the heterotopic bone, as a potential source of bone-forming osteoblasts, often results in the appearance of new heterotopic bone formation. Several alternative sources for bone-forming cells are currently being investigated as a tool for the unraveling of the FOP HO mechanism. Dermal fibroblasts have been demonstrated by Micha et al. to be a source of cells with osteoblastic properties. They showed that the FOP-derived fibroblasts have a high osteogenic potential [19]. Another source of human primary cells are cells isolated from extracted teeth. Many FOP patients develop a locked jaw, making oral care extremely difficult. In some cases, tooth extraction is the only dental treatment possible. Additionally, teeth are sometimes extracted to provide extra space in the mouth. There have been no reports of HO in the sockets at the extraction sites. Some groups have made use of the cells from the discarded primary teeth (SHED cells) [7,20], showing higher osteogenic differentiation in the FOP cells compared to control cells. Our group has recently shown that periodontal ligament cells obtained from FOP patients after tooth extraction show both osteogenic- as well as osteoclastogenic-inducing capacities, as do PLF from healthy individuals [21,22,23,24]. Here, we used these cells to investigate the Activin-A-induced transcriptome differences between controls and FOP patients. This study explored for the first time RNA sequencing as an open-ended approach for non-biased identification of potential new differences in early gene expression between control and FOP fibroblasts, directly following Activin-A binding. Given the unique signaling by Activin-A via the mutated ACVR-1, as described in mouse models [11] and iPS cell approaches [12], we hypothesize that Activin-A will induce early transcriptomic differences specifically in primary cells from FOP patients.

## 2. Materials and Methods

### 2.1. Periodontal Ligament Fibroblasts

Periodontal ligament cells were retrieved from 6 extracted teeth from 5 FOP patients (3 female patients age 26, 28, and 46 years and 2 male patients, age 21 and 69 years) and 6 healthy control donors (4 females age 19–26 years, 2 males, age 23–27 years). All FOP patients carried the classical R206H mutation. Written informed consent was obtained from each participant, and researchers were not able to trace the origin of the material to any individual, as required by Dutch law. Extractions were performed at the Department of Oral and Maxillofacial Surgery and Oral Pathology, Amsterdam UMC, Location VUmc. No differences in bone healing between the FOP patients and the control group following tooth removal were reported. Periodontal ligament cells were isolated, as previously described [21,25,26]. Shortly, the periodontal ligament was scraped off the middle one third of the root, and cells were allowed to grow out of the tissue by culturing them in culture medium, which consisted of Dulbecco’s modified eagle medium (DMEM, Gibco BRL, Paisley, Scotland) supplemented with 10% FCS (HyClone, Logan, UT, USA) and 1% antibiotics: 100 U/mL penicillin, 100 µg/mL streptomycin, and 250 ng/mL amphotericin B (Sigma, St. Louis, MO, USA). Cells were propagated, and 3rd passage cells were frozen and stored in liquid nitrogen. All experiments were performed with 4th passage cells.

### 2.2. Cell Culturing and RNA Isolation

Cells were first allowed to attach by overnight culturing in 6-well plates at a density of 3.2 × 10^4^ cells/cm^2^ (3 × 10^5^ cells/well) in DMEM + 10% FCS + 1% antibiotics. The following day media were replaced with media without or with 50 ng/mL Activin-A (Sigma, St Louis, MO, USA). After 24 h, RNA was isolated using the RNeasy Mini kit (Qiagen, Hilden, Germany) following the manufacturer’s instructions. RNA yield and quality were measured with the Synergy multi-mode reader (Biotek, Winooski, VT, USA).

### 2.3. RNA Sequencing

Sample and library preparation was performed using the NEXTflex Rapid Directional mRNA-Seq kit (Bioo Scientific, Austin, TX, USA). Single-end 75 bp RNA sequencing was performed on the Illumina NextSeq500 (Illumina, San Diego, CA, USA) at the Wilhelmina Children’s Hospital in Utrecht. Reads were aligned to the human reference genome GRCh37 using STAR version 2.4.2a. Picard’s AddOrReplaceReadGroups (v1.98) was used to add read groups to the BAM files, which were sorted with Sambamba v0.4.5, and transcript abundances were quantified with HTSeq-count version 0.6.1p1 using the union mode. Subsequently, reads per kilobase million reads sequenced (RPKMs) were calculated with edgeR’s RPKM function. Differentially expressed genes were identified using the DESeq2 package with standard settings. In the first analysis, the genes with a non-false discovery rate corrected *p* value of <0.01 were used for the pathway and GO analysis. To finally assess on the single gene level which genes were statistically significant differentially expressed, we performed a false discovery correction of 10%, generating *p* adjusted (*p*(adj)) values. For these *p*(adj) values, we used a cutoff of 0.1 (*p*(adj) < 0.1) to determine significance.

### 2.4. Pathway and Gene Enrichment Analysis

Pathway analysis and functional enrichment analysis were performed on the differentially expressed genes with *p* < 0.01, using the GeneMania application version 2017-03-14 at www.genemania.org [27,28,29] using standard settings. The program builds a network based on the interactions between the input genes (e.g., genes upregulated by Avtivin-A in the FOP cells) and adds genes that are relevant to that network. The pathway maps shown in Figure 2 are an adjustment to the enrichment map consolidated pathways described by Merico et al. [30]. Boxed genes are genes from the input list; the other genes are relevant genes added by GeneMania. Size of the nodes is dependent on the number of interactions that gene has with the network. The gene enrichment analysis was performed with the ToppGene suite application ToppFun version September 2020 [31,32] using standard settings.

## 3. Results

### 3.1. Different Donors Cluster Together

**Figure 1:**
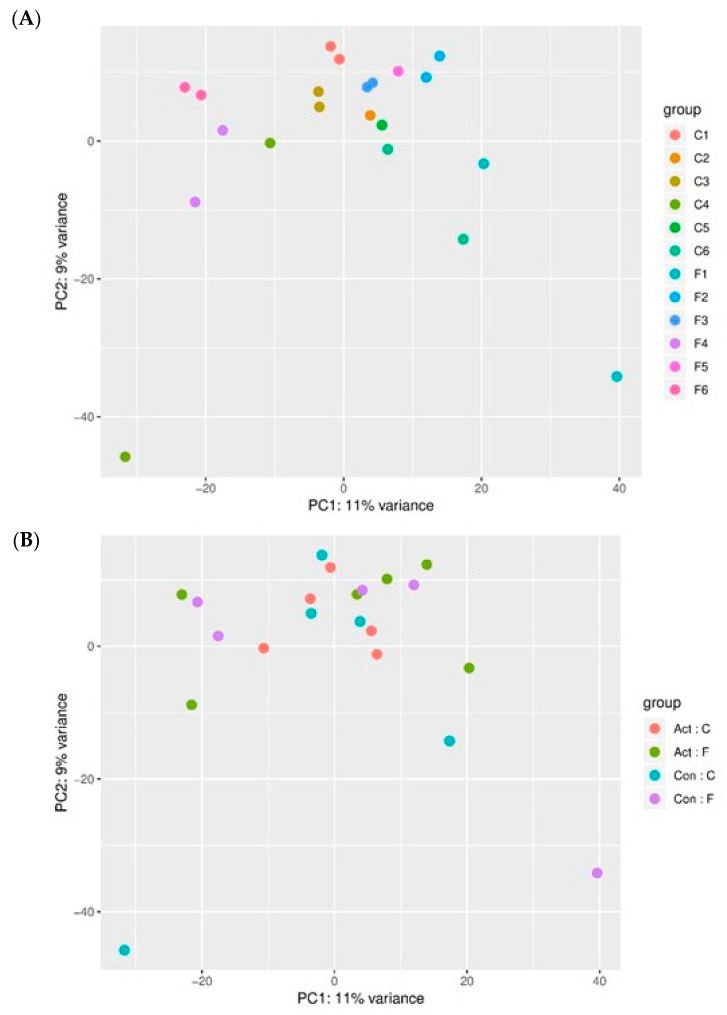
Donors group together. (**A**) PCA plot depicting inter-donor variability; (**B**) PCA plot depicting inter-experimental variability. Non-supervised principal component analysis shows that donors tend to group together, indicating that the variation between the control and FOP samples is larger than the comparison between control and experimental conditions. Inter-donor variability is shown in Figure 1A; each control and each FOP sample is represented by two same-colored dots, one indicating the control condition and one indicating the experimental condition (+Activin-A). Inter-experimental variability is shown in Figure 1B, each experimental condition is depicted by dots of the same color. Act:C = Control samples with Activin-A, ACT:F = FOP samples with Activin-A, Con: C = Control samples without Activin-A, Con:F = FOP samples without Activin-A. Samples Control 2 with Activin-A, Control 5 without Activin-A, and FOP 5 without Activin-A did not pass the QC filters and were not used in these and subsequent analysis.

To compare the transcription profiles of the different donors, we first performed non-supervised Principal Component Analysis (PCA) of all samples that passed the QC filters. Two control libraries and one FOP (Control 2 + Activin-A, Control 5–Activin-A and FOP 5–Activin-A) library did not pass the Quality Control filter, and therefore these samples were excluded from further statistical analysis. Figure 1A depicts the inter-donor variability between the samples. Figure 1B depicts the variability induced by the experimental conditions (e.g., without or with Activin-A). This analysis shows that the two culture conditions of one donor tend to cluster together, indicating that the variability between donors is higher than the variability induced by the Activin-A treatment.

### 3.2. Activin-A Activates Distinct Pathways in FOP Cells

**Figure 2:**
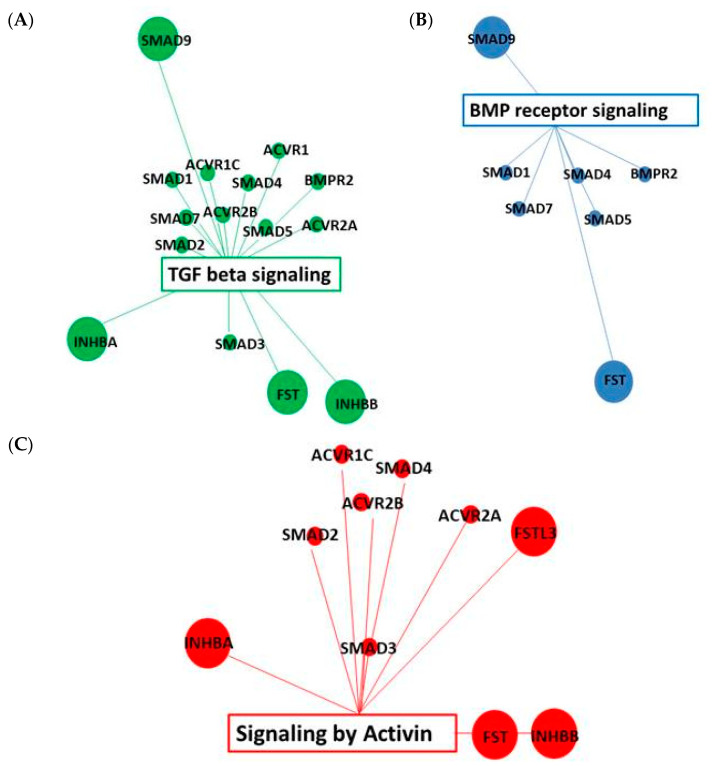
TGF-beta, BMP, and Activin signaling pathways are associated with upregulated genes by Activin-A. (**A**) Association with TGF-beta signaling pathway; (**B**) Association with BMP receptor signaling pathway. (**C**) Association with Activin signaling pathway. Pathway analysis on non-FDR-corrected differentially expressed genes with *p* < 0.01 performed with GeneMania (genemania.org, accessed on 21 September 2020). Figures are adjustments of the enrichment map of consolidated pathways TGF-beta signaling (MSigDB M2642) (**A**), BMP receptor signaling (MSigDB M181) (**B**), and signaling by Activin (MSigDB M26965) (**C**), Boxed genes are genes from the input list; the other genes are relevant genes added by GeneMania. Size of the nodes is dependent on the number of interactions that gene has with the network.

We next investigated which genes were influenced by Activin-A in the PLF cells. Using the non-FDR corrected gene expression data with *p* < 0.01, we found that 131 genes were differentially expressed in FOP cells (69 upregulated and 62 downregulated genes) as compared to 46 genes in the control cells (18 upregulated and 28 downregulated genes), see supplementary Tables S1 and S2. Network analysis using the GeneMania application showed that the genes upregulated by Activin-A in the FOP cells show an association with different pathways involved in, among others, TGF-beta signaling (MSigDB M2642) (Figure 2A), BMP receptor signaling (MsigDB M181) (Figure 2B), and signaling by Activin (Mysid M26965) (Figure 2C) showing Activin-A indeed induces its known signaling pathways also in this primary cell system. Additionally, functional enrichment analysis performed in GeneMania showed gene ontology terms enriched among the upregulated genes. These GO terms are involved in Activin, TGF-beta, and BMP signaling; the top 10 of the associated GO terms are listed in Table 1. None of these pathways or GO terms were found to be associated to Activin-A stimulation in the control cells (data not shown), indicating that Activin-A signals distinctly differently in the FOP cells as compared to in the control cells. Gene enrichment analysis on the FOP cells using ToppGene Suite showed that the differentially expressed genes in biological domain molecular functio, are mainly associated with gene ontology (GO) terms linked to cytoskeletal and actin binding (Table 2). In the biological domain biological process, the GO terms are linked to cell–cell and cell–substrate junctions and responses to injury and endogenous stimuli (Table 3). In the cellular component domain, the genes are associated with focal adhesions and cell–cell and cell–substrate junctions (Table 4). The observed association with the collagen gene family is possibly also related to bone metabolism (Table 5). None of these associations were found in the control cells.

### 3.3. Activin-A Induces Differential Gene Expression in FOP Cells

**Figure 3:**
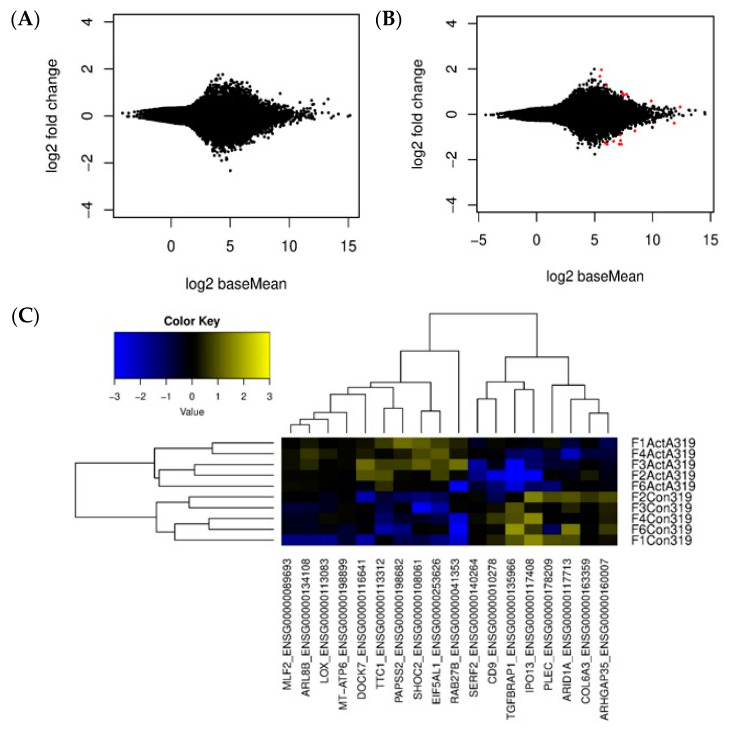
Activin-A induces differential gene expression only in FOP cells. Graphic display of differential gene expression (MA plot) in which the Log2 Fold Change induced by Activin-A treatment is plotted against the mean of normalized counts. Differentially expressed genes (*p*(adj) < 0.1) depicted as red dots. (**A**) MA plot of control samples shows no differentially expressed genes. (**B**) MA plot of FOP shows some differential gene expression induced by Activin-A, *n* = 4 for control and *n* = 5 for FOP samples. (**C**) Gene expression heatmap from median centered supervised gene expression analysis of the differentially expressed genes in the FOP cells. (*p*(adj) < 0.1, Z score is Log2 scale), *n* = 5.

Subsequent analysis on individual gene expression level was performed only on samples with both libraries, without and with Activin-A, per donor and corrected for donor variances in the final model. A false discovery correction of 10% was performed generating *p*(adj) values. Under these criteria, the final analyses were performed on 4 control samples and 5 FOP samples. To graphically display the differential gene expression induced by Activin-A, MA plots were generated where the Log2 fold change (M) was plotted against the average expression across the samples (A) using a *p*-adjusted cut-off value of 0.1. No differentially expressed genes were observed in control samples (Figure 3A). Interestingly, using these same settings, differential gene expression was observed in the FOP samples, shown in the MA plot as red dots (Figure 3B).

### 3.4. Differential Gene Expression Is Induced by Activin-A in FOP Cells

Having demonstrated that Activin-A alters gene expression only in FOP cells, we next investigated which specific genes were differentially expressed under the influence of Activin-A in these cells. Median-centered supervised gene expression analysis showed distinct clusters of up- and downregulated genes in the untreated (−ActA) and Activin-A treated (+ActA) groups (Figure 3C). From this analysis, using a false detection rate (FDR) of 10%, a list of the highest up- and downregulated genes was constructed as shown in Table 6. The genes that are more than two-fold upregulated by Activin-A in the FOP cells are somehow involved in bone metabolism. For instance, SHOC2 is a positive regulator of the MAP/ERK pathway, possibly involved in pathologies with bone and skeletal defects [33,34]. TTC1 activates RAS signaling, which regulates osteoprogenitor cell proliferation [35,36]. PAPSS2 is needed for sulfation of extracellular matrix molecules during bone development; deficiencies result in osteochondrodysplasias [37,38]. DOCK7 is a guanidine nucleotide exchange factor. Misty mice, with a loss of function mutation in this gene, show uncoupled bone remodeling and reduced bone formation probably linked to reduced brown fat production [39,40]. LOX is involved in the crosslinking of collagen and elastin and knock-out mice show decreased osteoblast differentiation [41,42]. A link to more direct Activin-A signaling can be found in RAB27B, which is involved in pituitary hormone secretion like FSH, the secretion of which is stimulated by Activin-A [43,44].

Except for COL6A3, the 1.3- to 3.9-fold downregulated genes seem to be less clearly associated with muscle and bone metabolism. COL6A3, which encodes for the alpha3 chain of collagen typeVI, is associated with the extracellular matrix of skeletal muscle, skin, and cartilage [45,46]. PLEC is an important molecule in muscle fibers [47]. CD9 is involved in cell fusion processes, as seen in osteoclast formation and muscle cell fusion [48,49]. TGFBRAP1, finally, is a Smad4 chaperone that associates with inactive TGFβ and Activin receptor complexes [50].

## 4. Discussion

The discovery of the causative mutation in the *ACVR1* gene resulting in the altered responsiveness of this BMP receptor [5] has paved the way to investigating the exact mechanism that underlies the heterotopic ossification seen in FOP. Especially during the last decade, great progress has been made, and the discovery of Activin-A as an activator of the mutated ACVR1 receptor [11,12] has stimulated the scientific FOP community to explore new therapeutic avenues. Here, we investigated the effect on early transcriptome differences induced by Activin-A in our recently established human primary FOP cell culturing system using periodontal ligament fibroblasts [21].

Our data show that indeed the mutated ACVR1 receptor has an altered responsiveness to Activin-A as compared to the non-mutated receptor. Initial gene expression analysis without false discovery rate correction showed that, when using a *p*-value cutoff of *p* < 0.01, 131 genes are differentially expressed in FOP cells as compared to 46 genes in the control cells. Pathway analysis of these genes using GeneMania shows an association with pathways involved in Activin, BMP, and TGFβ signaling only in the FOP cells. FOP is an autosomal dominant genetic disease, meaning that both the non-mutated as well as the mutated gene is being expressed, which has been demonstrated in FOP derived SHED cells and monocytes [22,51]. The association with the Activin and TGFβ signaling pathways indicates signaling via the non-mutated receptor, whereas the BMP signaling via SMAD1, 5, and 9 shows that the altered responsiveness of the mutated receptor to Activin-A as described by other groups [20,52] is also present in the periodontal ligament fibroblasts from FOP patients. Additionally, the gene enrichment analysis on these genes show a stronger reaction of the mutated receptor when exposed to Activin-A. There is an association with different GO terms, whereas no such association could be found in the control cells. HO in FOP often occurs after inflammation or injury [3], a process that is possibly mediated by an increase in Activin-A [3,11]. The altered gene expression profile seen here in the FOP cells after short exposure to Activin-A shows an association with GO terms GO 0009611 (response to wounding), GO:0009719 (response to endogenous stimulus), and GO:0071495 (cellular response to endogenous stimulus). This probably reflects the altered reaction described in FOP after injury or infection [53,54,55]. Under normal circumstances when Activin-A binds to the non-mutated ACVR1 receptor, it acts as an inhibitor of the canonical BMP-mediated signaling via ACVR1 and subsequent osteogenic differentiation. In FOP, however, the mutation results in activation of the mutated ACVR1 upon Activin-A binding, resulting in enhanced osteogenic differentiation. The association with the Activin-A-induced upregulated genes in the FOP cells with the collagen gene family observed in this study probably reflects the osteogenesis process taking place as a response to this altered signaling of the mutated ACVR1.

The inter-donor variability has a higher impact on the difference in overall expression compared to the experimental condition (e.g., without or with Activin-A), as described in Figure 1, suggesting that differences on the single-gene-expression level induced by Activin-A treatment could be difficult to detect above the “noise” induced by the above-mentioned inter-donor variability. Surprisingly, despite this fact, supervised gene expression analysis using a false discovery rate of 10% showed that differential expression was seen, but only in the FOP cells. Some of the genes that showed a higher than two-fold upregulation in the FOP cells under the influence of Activin-A can be related to bone metabolism or heterotopic ossification and can thus be regarded as potential targets to inhibit HO in FOP. These genes will be discussed next in the context of the process of endochondral bone formation.

Heterotopic ossification, as seen in FOP, is believed to be formed via endochondral bone formation where chondrogenic differentiation of stem cells results in cartilage formation that is subsequently converted to bone. We find an increase in DOCK7 expression in the FOP cells, possibly resulting in increased chondrogenesis and osteogenesis, ultimately resulting in enhanced HO. The mutated ACVR1 has been shown to play an important role in enhanced chondrogenic differentiation in a gain-of-function mouse model [56]. In mouse models of HO, one of the early steps in the formation of HO is the biogenesis of brown adipose tissue (BAT) [57]. This BAT creates a hypoxic environment that is suggested to induce chondrogenic differentiation of mesenchymal stem cells [58] and subsequent endochondral bone formation. Misty mice lack BAT and show decreased osteogenesis and increased osteoclast activity [40]. This uncoupling of bone formation and bone resorption is the result of a loss of function mutation in the *DOCK7* gene [39,59]. Additionally, the 2.3-fold upregulation of PAPPS2 observed in the FOP cells here may relate to the increased early stage endochondral ossification. The cartilage matrix that is produced during the early stages of endochondral ossification contains high concentrations of sulfate groups bound to several extracellular matrix proteins. PAPPS2 is a phosphosulphate phosphatase that catalyzes the sulfation of these extracellular matrix proteins [38]. Expression of PAPPS2 is partly regulated by TGFβ [60]. Mutations in PAPPS2 result in different types of osteochondrodysplasias, examples of which are described by Wang et al. [37]. The upregulation of TTC1 expression suggests that the RAS signaling pathway, which belongs to the osteogenic differentiation program, is activated. Osteogenic differentiation may take place via different pathways. One of the signaling pathways involved in osteogenic differentiation is the RAS signaling [61,62]. TTC1 is an adaptor protein that binds to Gα proteins, resulting in an interaction with small GTPase RAS that activates the RAS signaling pathway [35,36]. Similarly, the higher expression of SHOC2 seen here is an implication of increased RAS-MAPK signaling. SHOC2 is a leucine-rich repeat scaffolding protein that is involved in the RAS-MAPK signaling [33,34]. Mutations in this gene are linked to RASopathies like Mazzaniti syndrome and Noonan syndrome [63,64], both of which result in reduced postnatal growth. At a later stage of endochondral bone formation, the formation of collagen cross links is an important step that determines the integrity and strength of the bone. One of the enzymes involved in the formation of these crosslinks is Lysil Oxidase (LOX), a copper-dependent enzyme that catalyzes the deamination of lysine and hydroxylisine residues, thereby initiating the crosslinking of collagen molecules [41,42,65,66]. LOX-deficient mice show lower osteoblast differentiation and activity [42], and lower numbers of crosslinks are related to bone fragility in, for instance, aging and osteoporosis [65]. Interestingly, expression of LOX is partly regulated by the hypoxia-induced transcription factor HIF1 [67,68]. HIF1 is, together with Activin-A, upregulated during injury [20,53], providing potential links between hypoxia, FOP, and LOX expression.

Taken together, the upregulated genes described above play a role in either the early, chondrogenic, stages of endochondral bone formation (*DOCK7* and *PAPPS2*) or the later, more osteogenic, stages of this process (*TTC1*, *SHOC2.* and *LOX*). This indicates that the altered responsiveness of the mutated ACVR1 gene to Activin-A could induce heterotopic bone formation. Further studies to elucidate the exact role of athe bove-described genes in HO in FOP cells need to be performed. In these studies, blocking experiments with, for instance, follistatin will be included. We and other groups have previously shown that follistatin indeed inhibits the effect of Activin-A on other FOP derived primary cells [20,22]. We did not include such blocking experiments in this RNA sequencing experiment, because we first wanted to investigate which genes were influenced in the first place. Additionally, the subsequent data and statistical analysis where we would compare four different experimental conditions would become rather complex.

As discussed above, the up- and downregulated genes were identified only in FOP patient-derived cells. Nevertheless, and strikingly, the fold-change was relatively small, between 1.3- and 2.5-fold changes in the upregulated genes and 1.3- and 3.9-fold changes in the downregulated group. This suggests that the advanced heterotopic ossification in FOP could be the result of additive gene expression effects, rather than being caused by one dominantly over- or under expressed gene. QPCR experiments did not confirm the statistical differences, as seen in the RNAsequencing data analysis. This could be due to the fact that, as stated in Figure 1, the inter-donor variability is even higher than the differences induced by the experimental condition (e.g., addition of Activin-A), but probably more importantly, the fact that the average expression differences induced by Activin-A were below two-fold. In our experience, it is hard to reach statistical differences in QPCR experiments on human material with a low *n* as used here. In this paper, we use the periodontal ligament fibroblast as a cell biological tool to study early effects of Activin-A on osteogenic differentiation. Different groups have previously shown the osteoblast-like properties of these cells, making them a relatively accessible source of cells to study osteogenic differentiation in, for instance, FOP research [69,70,71,72]. However, as previously stated, following tooth extraction, no HO formation in the socket has been reported. This may imply that the periodontal ligament fibroblasts exhibit HO formation inhibiting properties, which may be the underlying reason for the relatively small fold changes witnessed, though an alternative possibility is the short incubation time with Activin-A. Longer exposure to the molecule might reveal more and possibly stronger effects on the transcriptome, resulting, when using osteogenic medium, in Activin-A-enhanced osteogenesis, as recently shown with tooth-associated cells by Wang et al. [20]. Gene enrichment and pathway analysis did not identify many or any new pathways associated to Activin-A signaling through the mutated receptor. This may be due to the relatively small number of differentially expressed genes, which does not allow for extensive pathway analysis. Nevertheless, when applying supervised gene expression analysis with a false discovery rate of 10%, our data show that Activin-A only induces differential gene expression in the FOP cells bearing the R206H mutation. The highest upregulated genes have all been linked to bone metabolism, and some even to heterotopic ossification in general, but none of these genes have, to our knowledge, previously been described to be linked to HO in FOP. The differential expression of genes somehow involved in bone formation by Activin-A exclusively in cells carrying the FOP mutation is in strong support of the therapeutic treatment rationale of inhibiting Activin-A. This study adds evidence to the notion that when disarming Activin-A’s malevolent effect, heterotopic bone formation can be tempered in the lives of people who suffer the daily anxiety of progressive ectopic bone formation with all its disabling consequences.

## Figures and Tables

**Table 1:** GO terms associated with upregulated genes by Activin-A in FOP cells.

ID	Function	FDR B&H	Coverage
GO:0032924	Activin receptor signaling pathway	8.03 × 10^−17^	12/31
GO:0007178	transmembrane receptor protein/threonine kinase signaling pathway	4.06 × 10^−14^	18/218
GO:0005072 *	transforming growth factor beta receptor, cytoplasmic mediator activity	2.25 × 10^−11^	7/10
GO:0090092	regulation of transmembrane receptor protein/threonine kinase signaling pathway	1.25 × 10^−10^	13/132
GO:0030509	BMP signaling pathway	2.19 × 10^−10^	11/79
GO:0032925	regulation of activin receptor signaling pathway	2.90 × 10^−9^	7/18
GO:0090100	positive regulation of transmembrane receptor protein/threonine kinase signaling pathway	2.53 × 10^−7^	8/53
GO:0060393	regulation of pathway-restricted SMAD protein phosphorylation	4.32 × 10^−7^	7/35
GO:0048185	Activin binding	7.92 × 10^−7^	5/10
GO:0060389	pathway-restricted SMAD protein phosphorylation	7.92 × 10^−7^	7/39

Top 10 GO terms enriched among the upregulated genes. Non-supervised functional enrichment analysis of GO terms associated with differentially upregulated genes by Activin-A in the FOP cells with *p* < 0.01 using GeneMania with default Benjamini and Hochberg’s False Discovery Rate (FDR B&H) settings. * GO:0005072 is replaced by GO:0000981.

**Table 2:** Molecular functions regulated by Activin-A in FOP cells.

ID	Name	FDR B&H	Genes from Input	Genes in Annotation
GO:0044877	protein-containing complex binding	3.60 × 10^−3^	23	1356
GO:0008092	cytoskeletal protein binding	4.73 × 10^−2^	17	1061
	actin filament binding			
GO:0051015	cell adhesion molecule binding	4.73 × 10^−2^	7	215
	actin binding			
GO:0050839	protein-containing complex binding	4.73 × 10^−2^	11	525
GO:0003779	cytoskeletal protein binding	4.73 × 10^−2^	10	451
	actin filament binding			

Non-supervised gene enrichment analysis of molecular function associated differentially expressed genes in the FOP cells with *p* < 0.01 using ToppGene suite using a Benjamini and Hochberg’s False Discovery Rate (FDR B&H) of 5%.

**Table 3:** Biological processes regulated by Activin-A in FOP cells.

ID	Name	FDR B&H	Genes from Input	Genes in Annotation
GO:0034330	cell junction organization	2.48 × 10^−2^	10	309
GO:0034329	cell junction assembly	2.48 × 10^−2^	9	245
GO:0007044	cell-substrate junction assembly	2.48 × 10^−2^	6	103
GO:0009611	response to wounding	2.48 × 10^−2^	15	756
GO:0009719	response to endogenous stimulus	2.80 × 10^−2^	25	1834
GO:0071495	cellular response to endogenous stimulus	3.54 × 10^−2^	22	1541
GO:0045196	establishment or maintenance of neuroblast polarity	3.54 × 10^−2^	2	3
GO:0045200	establishment of neuroblast polarity	3.54 × 10^−2^	2	3

Non-supervised gene enrichment analysis of biological processes associated differentially expressed genes in the FOP cells with *p* < 0.01 using ToppGene suite using a Benjamini and Hochberg’s False Discovery Rate (FDR B&H) of 5%.

**Table 4:** Cellular components regulated by Activin-A in FOP cells.

ID	Name	FDR B&H	Genes from Input	Genes in Annotation
GO:0005925	focal adhesion	6.75 × 10^−8^	17	411
GO:0030055	cell–substrate junction	6.75 × 10^−8^	17	421
GO:0005912	adherens junction	4.90 × 10^−7^	18	560
GO:0070161	anchoring junction	5.55 × 10^−7^	18	575
GO:0030054	cell junction	5.46 × 10^−7^	24	1352
GO:0030424	axon	1.88 × 10^−7^	14	817
GO:0030426	growth cone	2.16 × 10^−2^	7	226
GO:0030427	site of polarized growth	2.16 × 10^−2^	7	231
GO:0030496	midbody	2.82 × 10^−2^	6	177
GO:0005911	cell–cell junction	3.08 × 10^−2^	10	506
GO:0150034	distal axon	3.62 × 10^−2^	9	432

Non-supervised gene enrichment analysis of differentially expressed genes in the FOP cells with *p* < 0.01 using ToppGene suite using a Benjamini and Hochberg’s False Discovery Rate (FDR B&H) of 5%.

**Table 5:** Gene families regulated by Activin-A in FOP cells.

ID	Name	FDR B&H	Genes from Input	Genes in Annotation
939	Plakins	2.73 × 10^−2^	2	8
490	Collagens	2.73 × 10^−2^	3	46
634	Low density lipoprotein receptors	2.73 × 10^−2^	2	13
1149	NADH:ubiquinone oxidoreductase core subunits	2.73 × 10^−2^	2	14
596	Armadillo repeat containing|Importins	3.63 × 10^−2^	2	18

Non-supervised gene enrichment analysis of differentially expressed genes in the FOP cells with *p* < 0.01 using ToppGene suite using a Benjamini and Hochberg’s False Discovery Rate (FDR B&H) of 5%.

**Table 6:** (**A**) Upregulated genes by Activin-A in FOP cells. (**B**) Downregulated genes by Activin-A in FOP cells.

**A**			
**ENSEMBL ID**	**Gene Name**	**Fold Change**	***p*(adj)-Value**
ENSG00000041353	*RAB27B*	2.50	0.032
ENSG00000253626	*EIF5AL1*	2.50	0.098
ENSG00000108061	*SHOC2*	2.49	0.052
ENSG00000113312	*TTC1*	2.40	0.099
ENSG00000198682	*PAPSS2*	2.28	0.032
ENSG00000116641	*DOCK7*	2.23	0.098
ENSG00000134108	*ARL8B*	1.88	0.098
ENSG00000089693	*MLF2*	1.66	0.050
ENSG00000113083	*LOX*	1.47	0.060
ENSG00000198899	*MT-ATP6*	1.31	0.032
**B**			
**ENSEMBL ID**	**Gene Name**	**Fold Change**	***p*(adj)-Value**
ENSG00000163359	*COL6A3*	−1.25	0.074
ENSG00000178209	*PLEC*	−1.50	0.009
ENSG00000010278	*CD9*	−1.80	0.100
ENSG00000140264	*SERF2*	−1.85	0.089
ENSG00000160007	*ARHGAP35*	−1.90	0.032
ENSG00000117713	*ARID1A*	−2.42	0.098
ENSG00000117408	*IPO13*	−3.19	0.060
ENSG00000135966	*TGFBRAP1*	−3.90	0.021

Top 18 up and downregulated genes using supervised gene expression analysis. *p*(adj)-value are the *p*-values adjusted for an FDR of 10%.

## Data Availability

The RNAseq data presented in this study are available upon reasonable request. The data are not publicly available, because EU regulations do not allow us to put genetic or private data on the public domain (like GEO).

## Supplementary Materials

The following are available online at https://www.mdpi.com/article/10.3390/biomedicines9060629/s1, Table S1A: Non-FDR corrected Upregulated genes by Activin-A in Control cells; Table S1B: Non-FDR corrected Downregulated genes by Activin-A in Control cells; Table S2A: Non-FDR corrected Upregulated genes by Activin-A in FOP cells; Table S2B: Non-FDR corrected Downregulated genes by Activin-A in FOP cells.
