# Supplementary material for: Activin-A Induces Early Differential Gene Expression Exclusively in Periodontal Ligament Fibroblasts from Fibrodysplasia Ossificans Progressiva Patients
Source: Biomedicines. 2021 Jun 1;9(6):629. doi: 10.3390/biomedicines9060629 (PMC8229991; doi:10.3390/biomedicines9060629)
Supplement: Supplementary file 1 [file biomedicines-09-00629-s001.zip › biomedicines-1166319-supplementary.pdf]

biomedicines

MDPI

Supplementary

Table S1A Non-FDR corrected Upregulated genes by Activin-A in Control cells.

Ensembl_id	Gene_Name	Fold Ghange	*p* value
ENSG00000110925	*CSRNP2*	5.02	0.000
ENSG00000196505	*GDAP2*	3.72	0.001
ENSG00000100523	*DDHD1*	3.62	0.001
ENSG00000171466	*ZNF562*	3.60	0.001
ENSG00000058804	*NDC1*	3.44	0.002
ENSG00000162624	*LHX8*	3.21	0.003
ENSG00000131459	*GFPT2*	3.21	0.004
ENSG00000168769	*TET2*	2.94	0.007
ENSG00000175414	*ARL10*	2.91	0.008
ENSG00000134717	*BTF3L4*	2.88	0.008
ENSG00000089022	*MAPKAPK5*	2.85	0.009
ENSG00000087586	*AURKA*	2.84	0.008
ENSG00000068784	*SRBD1*	2.83	0.010
ENSG00000164211	*STARD4*	2.77	0.009
ENSG00000010017	*RANBP9*	2.69	0.006
ENSG00000074842	*MYDGF*	2.17	0.004
ENSG00000139278	*GLIPR1*	1.91	0.002
ENSG00000148848	*ADAM12*	1.69	0.002

Table S1B Non-FDR corrected Downregulated genes by Activin-A in Control cells.

Ensembl_id	Gene_Name	Fold Change	*p* value
ENSG00000198899	*MT−ATP6*	−1.58	0.003
ENSG00000103335	*PIEZO1*	−1.60	0.005
ENSG00000248527	*MTATP6P1*	−1.93	0.005
ENSG00000116786	*PLEKHM2*	−2.05	0.009
ENSG00000198695	*MT−ND6*	−2.13	0.007
ENSG00000078804	*TP53INP2*	−2.20	0.006
ENSG00000134697	*GNL2*	−2.21	0.009
ENSG00000167671	*UBXN6*	−2.38	0.001
ENSG00000144791	*LIMD1*	−2.50	0.006
ENSG00000185000	*DGAT1*	−2.54	0.007
ENSG00000169375	*SIN3A*	−2.60	0.005
ENSG00000086504	*MRPL28*	−2.67	0.009
ENSG00000124299	*PEPD*	−2.67	0.001
ENSG00000005175	*RPAP3*	−2.69	0.007
ENSG00000168411	*RFWD3*	−2.70	0.004
ENSG00000126821	*SGPP1*	−2.71	0.006
ENSG00000130299	*GTPBP3*	−2.77	0.008
ENSG00000103152	*MPG*	−2.82	0.006
ENSG00000149557	*FEZ1*	−2.84	0.005
ENSG00000188177	*ZC3H6*	−2.88	0.007
ENSG00000164039	*BDH2*	−2.89	0.006
ENSG00000137221	*TJAP1*	−2.90	0.005
ENSG00000128185	*DGCR6L*	−2.91	0.007
ENSG00000164687	*FABP5*	−2.96	0.006

ENSG00000146350	*TBC1D32*	−2.99	0.005
ENSG00000175063	*UBE2C*	−3.01	0.006
ENSG00000125703	*ATG4C*	−3.32	0.003
ENSG00000154642	*C21orf91*	−3.40	0.002

Non-FDR corrected differentially expressed genes with $p < 0.01$ in control cells.

Table S2.A Non-FDR corrected Upregulated genes by Activin-A in FOP cells.

Ensembl_id	Gene_Name	Fold Change	*p* value
ENSG00000100523	*DDHD1*	3.39	0.000
ENSG00000095485	*CWF19L1*	2.79	0.001
ENSG00000164815	*ORC5*	2.76	0.002
ENSG00000064651	*SLC12A2*	2.74	0.001
ENSG00000175283	*DOLK*	2.61	0.002
ENSG00000166925	*TSC22D4*	2.53	0.004
ENSG00000185619	*PCGF3*	2.52	0.002
ENSG00000162688	*AGL*	2.51	0.002
ENSG00000041353	*RAB27B*	2.50	0.000
ENSG00000253626	*EIF5AL1*	2.50	0.000
ENSG00000108061	*SHOC2*	2.49	0.000
ENSG00000120693	*SMAD9*	2.48	0.005
ENSG00000185418	*TARSL2*	2.47	0.005
ENSG00000168566	*SNRNP48*	2.46	0.005
ENSG00000204291	*COL15A1*	2.43	0.006
ENSG00000171204	*TMEM126B*	2.42	0.005
ENSG00000113312	*TTC1*	2.40	0.000
ENSG00000187713	*TMEM203*	2.40	0.007
ENSG00000141219	*C17orf80*	2.36	0.008
ENSG00000085721	*RRN3*	2.34	0.008
ENSG00000173193	*PARP14*	2.32	0.008
ENSG00000105849	*TWISTNB*	2.32	0.009
ENSG00000234741	*GAS5*	2.32	0.007
ENSG00000106012	*IQCE*	2.31	0.004
ENSG00000156256	*USP16*	2.30	0.003
ENSG00000157110	*RBPMS*	2.30	0.002
ENSG00000080802	*CNOT4*	2.28	0.009
ENSG00000198682	*PAPSS2*	2.28	0.000
ENSG00000116641	*DOCK7*	2.23	0.000
ENSG00000136603	*SKIL*	2.14	0.003
ENSG00000135446	*CDK4*	2.13	0.006
ENSG00000114450	*GNB4*	2.13	0.001
ENSG00000165525	*NEMF*	2.10	0.008
ENSG00000114346	*ECT2*	2.10	0.002
ENSG00000066044	*ELAVL1*	2.08	0.010
ENSG00000035681	*NSMAF*	2.07	0.008
ENSG00000010404	*IDS*	1.96	0.008
ENSG00000065809	*FAM107B*	1.94	0.002
ENSG00000157680	*DGKI*	1.94	0.004
ENSG00000163629	*PTPN13*	1.88	0.002
ENSG00000134108	*ARL8B*	1.88	0.000
ENSG00000111817	*DSE*	1.85	0.001
ENSG00000116199	*FAM20B*	1.84	0.005

ENSG00000174695	*TMEM167A*	1.83	0.008
ENSG00000138095	*LRPPRC*	1.80	0.003
ENSG00000145495	*MARCH6*	1.78	0.004
ENSG00000186318	*BACE1*	1.78	0.001
ENSG00000104164	*BLOC1S6*	1.78	0.003
ENSG00000163069	*SGCB*	1.78	0.008
ENSG00000115760	*BIRC6*	1.77	0.002
ENSG00000162704	*ARPC5*	1.77	0.004
ENSG00000197111	*PCBP2*	1.73	0.008
ENSG00000102753	*KPNA3*	1.72	0.009
ENSG00000089693	*MLF2*	1.66	0.000
ENSG00000151239	*TWF1*	1.63	0.008
ENSG00000107863	*ARHGAP21*	1.60	0.005
ENSG00000089737	*DDX24*	1.56	0.006
ENSG00000096696	*DSP*	1.55	0.004
ENSG00000084733	*RAB10*	1.53	0.003
ENSG00000113083	*LOX*	1.47	0.000
ENSG00000152377	*SPOCK1*	1.39	0.007
ENSG00000140575	*IQGAP1*	1,38	0.001
ENSG00000167601	*AXL*	1,36	0.007
ENSG00000122641	*INHBA*	1,32	0.008
ENSG00000198899	*MT–ATP6*	1,31	0.000
ENSG00000198840	*MT–ND3*	1,29	0.006
ENSG00000134363	*FST*	1,27	0.001
ENSG00000166598	*HSP90B1*	1,27	0.007
ENSG00000198763	*MT–ND2*	1,20	0.006

Table S2.B Non-FDR corrected Downregulated genes by Activin-A in FOP cells.

Ensembl_id	Gene_Name	Fold Change	*p* value
ENSG00000142156	*COL6A1*	−1.20	0.008
ENSG00000100345	*MYH9*	−1.23	0.007
ENSG00000163359	*COL6A3*	−1.25	0.000
ENSG00000196924	*FLNA*	−1.26	0.002
ENSG00000123384	*LRP1*	−1.27	0.002
ENSG00000140526	*ABHD2*	−1.35	0.007
ENSG00000204262	*COL5A2*	−1.36	0.009
ENSG00000110880	*CORO1C*	−1.39	0.006
ENSG00000188229	*TUBB4B*	−1.45	0.003
ENSG00000174231	*PRPF8*	−1.45	0.008
ENSG00000167526	*RPL13*	−1.48	0.002
ENSG00000178209	*PLEC*	−1.50	0.000
ENSG00000198542	*ITGBL1*	−1.52	0.002
ENSG00000143947	*RPS27A*	−1.60	0.007
ENSG00000129103	*SUMF2*	−1.65	0.010
ENSG00000122642	*FKBP9*	−1.66	0.007
ENSG00000197226	*TBC1D9B*	−1.69	0.008
ENSG00000164587	*RPS14*	−1.71	0.002
ENSG00000125991	*ERGIC3*	−1.76	0.010
ENSG00000089159	*PXN*	−1.78	0.008
ENSG00000010278	*CD9*	−1.80	0.000
ENSG00000172053	*QARS*	−1.83	0.005

ENSG00000140264	*SERF2*	−1.85	0.000
ENSG00000182446	*NPLOC4*	−1.89	0.004
ENSG00000160007	*ARHGAP35*	−1.90	0.000
ENSG00000128245	*YWHAH*	−1.96	0.008
ENSG00000168066	*SF1*	−1.96	0.001
ENSG00000136193	*SCRN1*	−1.99	0.001
ENSG00000159461	*AMFR*	−2.00	0.002
ENSG00000120256	*LRP11*	−2.01	0.010
ENSG00000129245	*FXR2*	−2.05	0.006
ENSG00000063438	*AHRR*	−2.11	0.005
ENSG00000100297	*MCM5*	−2.13	0.004
ENSG00000079308	*TNS1*	−2.14	0.002
ENSG00000140181		−2.18	0.010
ENSG00000120063	*GNA13*	−2.19	0.001
ENSG00000167118	*URM1*	−2.21	0.008
ENSG00000042753	*AP2S1*	−2.21	0.005
ENSG00000105520	*LPPR2*	−2.23	0.003
ENSG00000105058	*FAM32A*	−2.25	0.002
ENSG00000108592	*FTSJ3*	−2.29	0.004
ENSG00000158122	*AAED1*	−2.31	0.010
ENSG00000165688	*PMPCA*	−2.35	0.003
ENSG00000117713	*ARID1A*	−2.42	0.000
ENSG00000092470	*WDR76*	−2.44	0.005
ENSG00000143774	*GUK1*	−2.44	0.001
ENSG00000116514	*RNF19B*	−2.45	0.006
ENSG00000115107	*STEAP3*	−2.47	0.002
ENSG00000257621	*PSMA3−AS1*	−2.48	0.004
ENSG00000068903	*SIRT2*	−2.54	0.003
ENSG00000010803	*SCMH1*	−2.56	0.004
ENSG00000108292		−2.64	0.002
ENSG00000111361	*EIF2B1*	−2.73	0.001
ENSG00000142634	*EFHD2*	−2.74	0.001
ENSG00000033011	*ALG1*	−2.80	0.002
ENSG00000064932	*SBNO2*	−2.83	0.001
ENSG00000116791	*CRYZ*	−2.87	0.001
ENSG00000099899	*TRMT2A*	−2.90	0.001
ENSG00000117408	*IPO13*	−3.19	0.000
ENSG00000135709	*KIAA0513*	−3.47	0.000
ENSG00000135966	*TGFBRAP1*	−3.90	0.000
ENSG00000174442	*ZWILCH*	−3.98	0.000

Non-FDR corrected differentially expressed genes with $p < 0.01$ in FOP cells.
